# Recent Advances in Multiplexed Wearable Sensor Platforms for Real-Time Monitoring Lifetime Stress: A Review

**DOI:** 10.3390/bios13040470

**Published:** 2023-04-11

**Authors:** Heena Kim, Jaeyoon Song, Sehyeon Kim, Suyoung Lee, Yejin Park, Seungjun Lee, Seunghee Lee, Jinsik Kim

**Affiliations:** Department of Biomedical Engineering, College of Life Science and Biotechnology, Dongguk University, Seoul 04620, Republic of Korea

**Keywords:** mental stress, multiplexed sensor, wearable sensor, physical biomarker, chemical biomarker

## Abstract

Researchers are interested in measuring mental stress because it is linked to a variety of diseases. Real-time stress monitoring via wearable sensor systems can aid in the prevention of stress-related diseases by allowing stressors to be controlled immediately. Physical tests, such as heart rate or skin conductance, have recently been used to assess stress; however, these methods are easily influenced by daily life activities. As a result, for more accurate stress monitoring, validations requiring two or more stress-related biomarkers are demanded. In this review, the combinations of various types of sensors (hereafter referred to as multiplexed sensor systems) that can be applied to monitor stress are discussed, referring to physical and chemical biomarkers. Multiplexed sensor systems are classified as multiplexed physical sensors, multiplexed physical–chemical sensors, and multiplexed chemical sensors, with the effect of measuring multiple biomarkers and the ability to measure stress being the most important. The working principles of multiplexed sensor systems are subdivided, with advantages in measuring multiple biomarkers. Furthermore, stress-related chemical biomarkers are still limited to cortisol; however, we believe that by developing multiplexed sensor systems, it will be possible to explore new stress-related chemical biomarkers by confirming their correlations to cortisol. As a result, the potential for further development of multiplexed sensor systems, such as the development of wearable electronics for mental health management, is highlighted in this review.

## 1. Introduction

With the advancement of biomedical technology, there has been a surge in research interest in measuring mental stress. Walter Cannon and Hans Selye [1,2,3] were the first to define stress, which has been linked to a variety of chronic and psychiatric diseases. Stress is an uncontrollable and unpredictable event that is triggered by external factors (environmental, physiological, and social) [4]. Moreover, internal factors such as disruptions to cognitive and immune systems, due to the frequent occurrence and severity of stress, can lead to illness [5,6,7,8]. As each individual is likely to encounter to a situation that causes stress, it is important to identify the stressor—the thing that causes stress—in order to manage stress in daily life.

The conventional methods for measuring stress are to assess the stress level using a questionnaire or to measure physiological signals quantitatively using physical tests. Measuring stress via a questionnaire has been widely used as a self-evaluation method for relatively long periods in various methods, such as the stress response inventory, the Beck anxiety inventory, and the perceived stress scale [9,10,11]. However, continuous stress monitoring is challenging because of the poor reliability in repetitive analysis, and it is significantly affected by individual conditions [12,13]. Additionally, stress cannot be measured in certain situations, such as during sleep. Conversely, measuring the short-term stress reactions via electrical signaling of physiological signals such as heart rate (HR) or body temperature is possible [14,15,16,17,18]. This method could efficiently measure the acute stress response that changes rapidly within a short time; however, long-term quantification with the measurement of the stress level is challenging, owing to factors such as the bulkiness of the system. Because of the low reliability and the technical limitations of these aforementioned conventional methods, the stress measurement results are cross-validated by measuring the concentration of chemical biomarkers, such as cortisol, in the body fluid for greater accuracy [19]. The change in the concentration of cortisol in the body fluid from baseline, which is different for each individual, can be used to secure higher reliability when tracking the intensity and frequency of stress [20]. Therefore, the use of body-fluid-based measurements of cortisol for stress monitoring has been studied extensively [21,22]. Moreover, chemical biomarkers are known to be more relevant for diagnosing health conditions than physical biomarkers, because they can directly reflect symptoms that manifest in our body [23]. However, as cortisol release occurs in a diurnal cycle, different concentrations are measured at different times; consequently, accurate stress measurement is demanding [24,25]. Additionally, only a significantly small number of stress-related chemical biomarkers with low concentrations can be observed without using invasive measurement techniques in body fluids. In the case of cortisol, the concentration ranges in sweat and blood are 8–140 ng/mL and 20–230 ng/mL, respectively [26,27]. In addition to cortisol, sweat contains neuropeptide Y, which is involved in biological processes related to stress, and has a concentration range of 50–200 pg/mL [28]. Due to the low concentration of these chemical indicators that makes it difficult to detect stress in sweat, it is challenging to develop a detection method with greater robustness [29]. This suggests that exploring new stress-related and sweat-based chemical biomarkers by identifying their correlations with representative stress-related biomarkers is demanding. Hence, wearable multiplexed sensors that can detect multiple biomarkers, such as different combinations of physical and chemical biomarkers, for more accurate and quantitative measurements of stress monitoring, are emerging [30].

**Figure 1:**
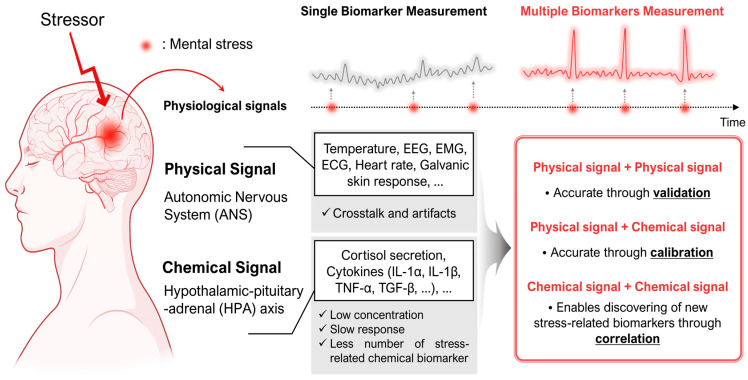
Mental stress triggered in response to the stressor and an illustration of the stress-related signals. Red box indicates the advantages of measuring multiple stress-related biomarkers.

This review focuses on recent wearable sensor studies that detect combinations of physical and chemical biomarkers for the development of mental stress-monitoring systems. The most recent research on multiplexed sensors is discussed based on wearable sensor research. Furthermore, the discovery of new stress-related chemical biomarkers through correlations with representative biomarkers is discussed. Multiplexed sensor systems for stress monitoring can lead to accurate stress diagnosis through validation or calibration, allowing for stressor identification and management. Figure 1 depicts examples of stress-related physical and chemical biomarkers, as well as the impact of measuring multiple biomarkers, i.e., physical and chemical biomarkers in combinations.

## 2. Physiological Effects of Stress

### 2.1. Physiological Response Path Caused by Stress

The central nervous system acts as a mediator of the stress response system, which could be categorized into the responses of the autonomic nervous system (ANS) and neuroendocrine system (NES), based on the response pathways involved [31,32]. The response of the ANS, also known as “fight or flight,” is caused by the sympathetic nervous system (SNS) [33,34]. The stress response of the SNS is predominantly represented by an immediate response in the peripheral organs (i.e., HR, body temperature, etc.); consequently, acute stress can be measured more easily with this response than with the response from the NES. In terms of the NES, the hypothalamic–pituitary–adrenal (HPA) axis is the center of the stress response system, and numerous neurotransmitters and hormones are released or controlled [35,36,37]. The stress response of NES is considerably slower and more sluggish than that of the ANS; therefore, it is beneficial for measuring chronic stress [31,38,39].

### 2.2. Physiological Biomarkers Related to Stress

The physical biomarkers and chemical biomarkers are examples of biomarkers, which are indicators that provide information about an individual’s health [26]. According to the aforementioned pathways of physiological response to stress, physical biomarkers are generated by the ANS, and chemical biomarkers are released through the NES or HPA axis. Thus, representative stress-related biomarkers, which are arranged based on the origin of their signals, in Table 1 are classified based on the type of biomarker and its generated pathways. These biomarkers are utilized and developed to monitor stress through measuring a single biomarker quantitatively.

Respiration, pulse rate, HR variation (HRV), photoplethysmograms, electrodermal activity (EDA), skin temperature (ST), and sweating rate are known as the physical biomarkers that humans perceive as a response to stress. In addition, the representative evaluation methods for measuring electrical signals generated by the body are electroencephalography (EEG), electrocardiography (ECG), or electromyography (EMG) [60].

An EEG measures brain activities generated through signals closely related to mental and physical stresses. EEG signals are sorted by frequency bands, consisting of the delta, theta, alpha, beta, and gamma waveforms, representing the different states of emotions [40,41]. When analyzing stress using an EEG signal, ‘feature extraction’ for the raw signal and ‘classification’ based on it are performed. Support vector machines (SVM), logistic regression (LR), naïve bayes (NB), and K-nearest neighbors (KNN) are common classifier algorithms for stress monitoring, and efforts to improve stress monitoring accuracy are ongoing [43,44]. The ECG records and extracts the electrical signal related to HRV and HR as a waveform, by measuring the time difference between the R peaks, which is the electrical signal that passes the ventricular walls and is observed in the signal derived from QRS complexes [61,62]. Since the waveforms seen in an ECG, including P-, Q-, R-, S-, and T-waves, represent different states of the heart, the R–R interval-based analysis is a traditional analysis, and recent attempts to investigate the use of other QRS complex parameters, such as the P–R interval and S–T interval, have been made [47]. The HRV, which represents the response to physiological and environmental stimuli through changes in the heartbeat, was primarily obtained from ECG; however, it is now detectable using wearable devices, enabling noninvasive detection and monitoring [45,46]. EMG evaluates the electrical activity of skeletal muscles, and can also be used as a stress marker. As it is known that facial and trapezius muscle tones are increased by mental stress, EMG has been used in polysomnography studies to analyze the stress level during sleep [48,49]. An EDA, also called galvanic skin response (GSR), is a measure of the changes in skin conductivity according to sweat secretion. An EDA is widely used to detect physiological stress levels [50,51]. In stressful situations, the conductivity of the skin increases, and this change can be used to monitor stress levels [63,64]. Tonic responses (skin conductivity level, SCL) and rapid changes comprise the EDA signal (skin conductivity reaction, SCR). SCL is the sympathetic system’s underlying activity and reflects long-term stress, whereas SCR reflects individual stimuli, such as momentary stress, and changes in events, such as cognitive and emotional responses, which cause activation of brain regions. As a result, stress is analyzed by extracting tonic and phasic responses from raw EDA data, and analyzing their phase and amplitude [52]. Stress causes changes in ST [53,54,55], and it is well-known that the amount of change varies, depending on the area or the object measured. When stress levels are measured using a physical biomarker, efforts are being made to improve accuracy through efficient data processing and algorithm-based analysis [43]. 

Nevertheless, the detection of stress through physical methods is a challenging task, owing to various limitations. For example, signal detection methods such as EEG, ECG, or EMG can generate systematic noise [42,65]. Dehydration of electrodes also increases the noise in high-impedance sites, thereby reducing the adherence of the electrode to a subject [66]. Due to the vulnerability to motion artifacts, position or movement (sitting, standing, or walking) can affect signal detection, disrupting mental stress measurements [51]. Moreover, similar to negative emotions, positive emotions also exhibit changes in the EMG [67]. Stress examination performed in controlled laboratory settings, where noise occurrence is suppressed, becomes inefficient when the detection is conducted in naturalistic settings, owing to hidden contexts in environmental conditions [68]. In addition to issues related to the accuracy, data based on comprehensive psychological signals, which were measured via ECG, EMG, EEG, and GSR measurements, showed continuous fluctuations at the minute scale from stress induction to recovery [69], indicating that the diagnosis of mental stress through physical methods can lower accuracy and reliability.

In the case of chemical biomarkers, cortisol, neuropeptide Y, and several cytokines (interleukin (IL)-1α, IL-1β, IL-6, IL-8, IL-22, tumor necrosis factor-α, and transforming growth factor-β) [70,71], which are secreted from eccrine glands, are the sweat analytes that can be detected as stress-related biomarkers [71,72,73]. These biomarkers enable a point-of-care platform, avoiding the mental stress that occurs during the sampling processes of invasive methods [74,75], and can be integrated with a wearable sensor system to analyze the stress response [59]. Among the various chemical analytes, cortisol is considered an attractive sweat biomarker, with its secretion depending on a diurnal circadian rhythm [24,25]. However, sweat detection remains challenging owing to several environmental impacts, such as variation in sweat volume, pH, temperature, humidity, and easy contamination by skin residue, which are responsible for differences in the sweat condition of an individual. These elements can degrade the performance of sweat cortisol detection [76,77,78]. Moreover, owing to the different cortisol concentrations at peak values, which vary based on human bodies [79], and cortisol’s blood-to-sweat lag [80], temporary detection and monitoring of a single analyte is considered insufficient for determining mental stress.

As a result, monitoring stress in real-time can be achieved by detecting numerous biomarkers, in addition to a single stress indicator. Multiple stress-related biomarkers can be used to study mental stress in order to take advantage of the complementary relationship between them and to reduce experimental error. There have been attempts to measure different biomarkers to address these issues, but the majority of them have been studied using many existing sensors or in arbitrarily demanding environments, rendering them inappropriate for monitoring life’s cumulative stress [81,82]. Therefore, monitoring stress response requires the deployment of patch- or wearable-type multiplexed sensors that may measure stress in a variety of people, more accurately and consistently, and it is anticipated that these will be a useful tool for correlation analysis, in order to find novel chemical biomarkers in addition to cortisol.

## 3. Multiplexed Sensor Systems for Stress Monitoring

In this section, the multiplexed sensors that detect the combinations of various physical biomarkers and chemical biomarkers are discussed. Table 2 is classified in the order of multiplexed physical sensors, physical–chemical sensors, and chemical sensors. Each multiplexed sensor is arranged terms of combinations applicable to monitor stress (referred to as applicability), and compared with the characteristics. The characteristics section explains how each multiplexed sensor works to improve performances by measuring multiple biomarkers. We selected the most representative multiplexed sensor systems based on the working principles—calibration, validation, and correlation—and we introduce them in Section 3.1, Section 3.2 and Section 3.3. Calibration is a working principle that can help prevent the impact on the measurement of each biomarker. Validation, also known as cross-validation, involves testing multiple biomarkers to achieve a specific result. This method is particularly useful when a single biomarker cannot provide the desired signal, as it could be helpful to identify new biomarkers while improving accuracy and performance. Correlation can also be utilized in terms of the limited number of multiplexed chemical sensors available, to discover new biomarkers apart from cortisol. Therefore, we introduce a multiplexed sensor system based on these three working principles in the following characteristics section.

### 3.1. Multiplexed Physical Sensor Systems

Stress detection using physical biomarkers, such as ECG, EMG, GSR, or respiration rate (RR), is currently being used in various ways [51,101,102,103]. However, using an individual biomarker to detect stress does not present an accurate value, because a single data type is likely to be affected by factors other than simple stress [51]. For example, the respiration-based method is not applicable in the case of a subject who performs voluntary breathing; in such cases, other parameters should be measured [104]. Therefore, detecting multiple biomarkers in a multiplexed sensor for stress monitoring is currently in the spotlight. A multiplexed physical sensor can achieve high accuracy through the validation of multiple data from each parameter, in addition to the real-time and long-term monitoring of stress.

**Figure 2:**
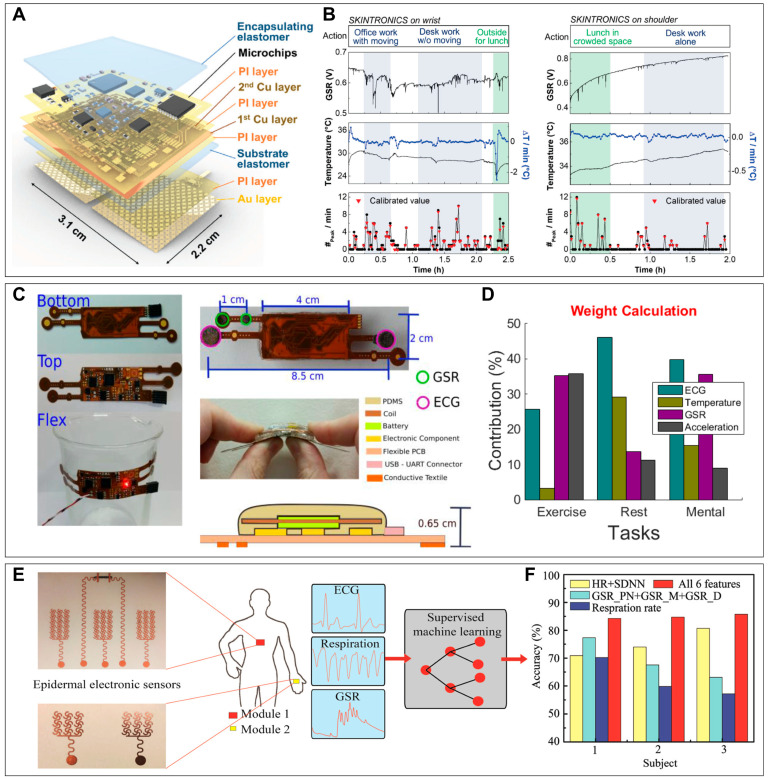
Multiplexed physical sensor systems. Stress monitoring through the acquisition and analysis of physiological signal data through the multiplexed physical sensor. (**A**) GSR and ST detection through SKINTRONICS and (**B**) stress analysis under various environments [83]. (**C**) The device, fabricated using a flexible printed circuit board to produce ECG and measure GSR; (**D**) its detection of various physiological signals when exercising, resting, and performing mental tasks, and their analysis for stress monitoring [84]. (**E**) The epidermal sensors that can produce ECG and measure RR and GSR, and (**F**) comparison of mental fatigue detection accuracy using multimodal sensors and a single sensor [85].

Kim et al. developed a fully integrated, stretchable, wireless skin-conformal bioelectronic (referred to as “SKINTRONICS”) that integrates soft, multilayered, nanomembrane sensors and electronics for continuous and portable stress monitoring, by monitoring both GSR and ST simultaneously in daily life (Figure 2A) [83]. A low-modulus elastomer that naturally adheres to the skin coats the device layer and serves as structural support. Experimentally, under mentally relaxed and stressed states, SKINTRONICS exhibits a greater ratio of identified stress peaks than conventional devices and a high signal-to-noise ratio (SNR) when standing or walking. Consequently, the device could detect stress accurately by measuring the number of GSR peaks per minute, which are calibrated based on temperature, because of the undesirable fluctuations in GSR caused by the changes in ST (Figure 2B). The device could detect GSR and ST for long-term stress monitoring in daily life and allows measurements for up to 7 h. Rosa et al. invented a flexible and multiplexed sensor system with textile electrodes that can be worn as a chest patch for monitoring mental health during extended periods [84]. The device consisted of a flexible printed circuit board layer with a conductive textile attached to the ECG and GSR channels, and was coated with polydimethylsiloxane (Figure 2C). The device can acquire three physiological signals: ECG, GSR, and body temperature. Stress validation performance was tested by comparing the response of the three different signals in different situations. Each signal has a different degree of reaction depending on the conditions of rest, exercise, and mental work (Figure 2D). As the existing method of using only one signal does not correctly distinguish between each condition, a multiplexed sensor system can more accurately analyze the degree of change in the value of each parameter, depending on the conditions the user is experiencing and, consequently, distinguish mental stress from other conditions. Yoon et al. demonstrated a human stress-monitoring patch with a small skin contact area and high flexibility to enhance comfort when wearing the patch [85]. This patch can measure three different physical biomarkers derived by ANS: ST, GSR, and arterial pulse wave, to detect stress. The performance of each sensor demonstrated a sensitivity of 0.31 Ω/°C, 0.28 µV, and a response time of 70 msec in the ST, skin conductance, and pulse wave sensors, respectively. The integrated multiplexed sensor system measures the psychological stress in the human physiological range by quantitatively and continuously analyzing stress.

Zeng et al. showed a multimodal epidermal electronic system for detecting ECG, RR, and GSR signals, as illustrated in Figure 2E, using machine learning algorithms to detect mental fatigue [85]. The main problem when measuring physical biomarkers is the vulnerability to daily movements, such as body motion. However, this group’s multiplexed sensor system exhibited considerable stability when measuring ECG signals under different conditions, such as yawning and compressing or stretching the body. In the case of compression and stretching, the sensor provided stable waveforms. Although the amplitude of the ECG signal decreased after being attached to the body for 24 h, an intact waveform was measured. Three physical biomarkers were extracted for validation and featured as the training data for machine learning. By utilizing these data, up to 89% accuracy can be obtained when machine learning is conducted for physiological data collected by the sensor (Figure 2F). Moreover, using these results, they were able to confirm the change in stress levels over time due to various mental-related activities. When machine learning was utilized, higher accuracy was achieved when integrating all the biomarkers, as compared to a single sensor. This shows the requirement for a multiplexed sensor system for stress monitoring. Kim et al. invented a stretchable hybrid electronic system (SHE) for physiological data monitoring, with wireless, soft, comfortable properties that obviate the need for skin preparation or electrolyte gels with aggressive adhesive properties [87]. SHE can produce ECG measurements and measure HR and RR, in order to detect the condition of a patient, such as the presence of mental stress. As mentioned above, ECGs can have respiratory artifacts; therefore, it can be utilized by considering HR and RR extractions. The SNR was higher in SHE than in commercial systems, and the two systems showed a high correlation when the data obtained from the two systems were compared, with slopes of 0.9760 for HR and 1.0545 for RR. Further, the SHE was used for long-term ECG monitoring for seven days, and demonstrated the advantages of real-time monitoring. Although SNR dropped by 3.3 dB over time, there were no significant changes of the ECG waveforms from day 1 to day 7, and the SNR was still measured at 18.2 dB. By using these parameters, real-time and accurate stress monitoring can be achieved.

Similar to the above research, commercial attempts are being made to precisely track the physical status on a wearable platform by carefully positioning various sensors, including those measuring GSR, HRV, BP, and skin temperature. Examples include the popular watches; Fitbit (Fitbit, San Francisco, CA, USA), Galaxy watch (Samsung, Suwon, Republic of Korea), and Apple watch (Apple, Cupertino, CA, USA). Additionally, the industry is constantly seeing the emergence of new patch-type products, such as Vivalink’s VitalScout. The need for multiplexed sensing to improve accuracy, and the market for wearable stress monitoring devices, are both expanding as these products are being developed regularly and new companies are entering the market.

### 3.2. Multiplexed Physical–Chemical Sensor Systems

The detection of multiple signals in terms of the physiological reactions to stress by a physical multiplexed sensor system helps in complementing two or more signals by cross-validation [105,106] and achieving greater accuracy [84] than when a single biomarker is detected in stress monitoring. Although this multiplexed physical sensor system is a promising device for monitoring stress, the complex signals derived by stress are not fully understood when a multiplexed sensor detects the same type of signal. When the HPA axis becomes dysfunctional as a result of chronic stress, brought on by prolonged exposure to stress [107], this results excessive and accumulated cortisol [39], which makes it difficult to identify acute stress and chronic stress when using multiplexed physical sensor. In the case of a multiplexed chemical sensor system, the signals of the chemical biomarkers are easily affected by heat sources [108], such as body temperature. Additionally, chemical biomarkers extracted from sweat have a sweat-to-blood lag [80] that causes a time delay, leading to the inaccurate monitoring of acute stress, resulting in difficulties in the treatment and elimination of stressors. This implies that collecting signals from various types of signals is necessary for stress monitoring with a comprehensive view. As there is only limited research on physical–chemical multiplexed sensor systems that can monitor mental stress, those that sense at least two different types of physiological signals are discussed in Section 3.2. In this paragraph, compensating for the limitations of individual signals and certain multiplexed physical–chemical sensor systems of several groups that can be applied to achieve a more comprehensive perspective on the monitoring of mental stress is mainly reviewed.

**Figure 3:**
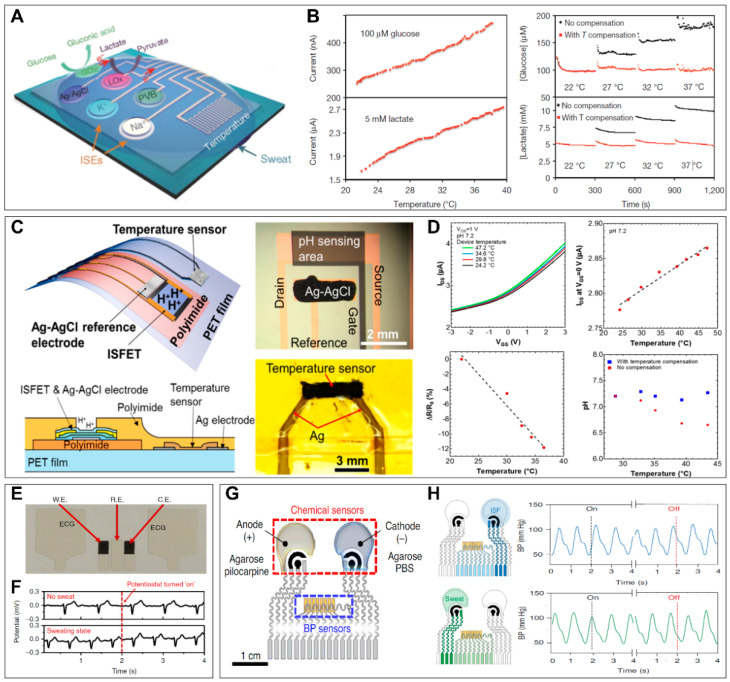
Multiplexed physical–chemical sensor systems. The compensation of one sensor by another sensor is shown. (**A**) Illustration of the hybrid sensor design, consisting of glucose, lactate, and temperature sensors, and the detection site of various biomarkers. (**B**) The graph (left) shows the change in current as temperature increases, on 100 µM of glucose and 5 mM of lactate. The other graph (right) shows the compensation of the chemical biomarkers according to the different temperature states [89]. (**C**) Description of the hybrid sensor containing temperature and pH sensors; (**D**) shows the calibrated results of pH by utilizing the temperature sensor [90]. (**E**) Actual sensor design of patch-type electrocardiography electrodes and the lactate sensor (comprising three electrodes that are linearly arranged at the center). (**F**) The graph (below the sensor design) shows the effect of applying a potentiostat to the heart rate measurements [91]. (**G**) The sensor’s design consists of chemical sensors for detecting glucose and lactate and physical sensors for detecting blood pressure (BP). (**H**) Graphs illustrating the ability of the sensor to eliminate the crosstalk between the applied voltage, which was used to detect the chemical biomarkers and the applied ultrasound that was used to detect the BP. The upper graph shows glucose–BP detection, and the lower graph shows lactate–BP detection [30].

Several research groups have developed multiplexed physical and chemical sensors consisting of a temperature sensor, which can be physically detected, to supplement the perspiration analysis of chemical biomarkers. Gao et al. developed a multiplexed wearable sensor by analyzing the biomarkers in sweat. The developed sensor simultaneously detects chemical biomarkers, including glucose and lactate, as well as the temperature (Figure 3A) [89]. This temperature sensor could not only analyze the ST, in order to measure the physiological conditions of an individual, but also eliminate the effect of temperature on the chemical biomarkers, i.e., glucose and lactate, as shown in Figure 3B. To enable long-term stress monitoring, they conducted on tests the sensors for up to five weeks to evaluate their stability. Each sensors showed less than 10% drift over the five weeks, indicating that stress monitoring in daily life is feasible. Similarly, Nakata et al. used a temperature sensor for compensating the limitations in pH detection, because pH potential is proportional to temperature according to the Nernst equation [90]. This multiplexed physical–chemical sensor is illustrated in Figure 3C. The effect of temperature change on pH is also shown in Figure 3D, with the pH value calibrated by utilizing the linearity of the temperature sensor.

Although measuring both physical and chemical biomarkers has a positive effect, such as supplementing the value for achieving accurate measurements related to stress, the multiplexed physical–chemical sensor system exhibits certain challenges when detecting the physiological signals, because the sensor requires an energy source for its operation as a module [109]; moreover, its energy source should not disturb the other types of sensors (which is referred to as crosstalk). Imani et al. designed a multiplexed physical–chemical sensor system by integrating an ECG electrode and a lactate sensor with negligible crosstalk. They also discussed the interference caused by the application of voltage to hybrid sensors, which can affect the ECG measurements (Figure 3E) [91]. Furthermore, they discussed the shunting effect, in which the electrically conductive medium shunts between the lactate sensor and the ECG electrodes, due to the ions that are present in sweat. They eliminated the shunting effect by creating a gap between each layer and adding a hydrophobic substrate. The geometry of the multiplexed physical–chemical sensor system and the real-time and simultaneous detection of HR are shown in Figure 3E,F, respectively.

Moreover, the correlation between chemical and physical biomarkers was analyzed by Sempionatto et al. [30]. They described a patch-type hybrid sensor for detecting glucose, lactate in interstitial fluid, sweat, and blood pressure to identify the unexplored correlation between chemical and physical biomarkers; their multiplexed sensor system was designed as shown in Figure 3G. They also demonstrated negligible crosstalk, which occurs when one sensor changes the signals of the other sensor (Figure 3H). Chen et al. developed wearable and flexible, organic, thin-film, transistor-based modules to monitor physical and mental stress, utilizing pH and HR sensors, respectively. However, this research is limited, as it does not integrate the modules or perform simultaneous measurements to detect mental stress; however, to the best of our knowledge, and based on the suggestions of this group in their proposed future works, a combination of both physical and chemical biomarkers can be applied for monitoring mental stress [110]. Hence, the real-time detection of both physical and chemical biomarkers can achieve a more comprehensive understanding of the physiological reactions related to mental health. This could lead to the removal of stressors, which are different in every individual; moreover, the management of these stressors can prevent the progression of stress-related chronic disease.

### 3.3. Multiplexed Chemical Sensor Systems

To reduce experimental errors in noninvasive stress measurements using chemical sensors, especially the biomarkers in sweat, two or more stress-related chemical biomarkers should be measured instead of measuring a single biomarker, as performed in conventional methods. Furthermore, to enable real-time monitoring, a patch- or wearable-type sensor must be developed. Conventional stress sensors are based on physical biomarkers, such as GSR, ST, and HR measurements. However, physical biomarkers are easily induced by non-stress-related causes, such as weather conditions and motions in daily life [111]. Therefore, there is a requirement for a chemical sensor that extracts and senses stress-related biomarkers in sweat. The stress biomarker in sweat with the greatest potential is cortisol, and there are many single sensors that measure cortisol in conventional studies. However, to improve the accuracy of stress monitoring, multiple chemical biomarkers that are related to cortisol need to be detected simultaneously. We propose that detecting biomarkers using various metabolic reactions associated with cortisol can identify new chemical biomarkers related to stress. This is because increased levels of cortisol influence the regulation of diverse physiological processes, such as glucose levels and carbohydrate metabolism [112]. In this paragraph, we described the multiplexed chemical sensors, with the potential to measure stress by detecting various stress-related chemical biomarkers.

Pali et al. developed wearable awareness through continuous hidrosis (WATCH) sensor to continuously monitor the biomarkers in sweat, i.e., cortisol and glucose [95]; the study identified that the HPA axis is activated because of stress, and the HPA axis stimulation leads to an increase in cortisol levels, which stimulates gluconeogenesis, leading to an increase in glucose levels. Another study revealed that chronic stress has evident effects on glucose metabolism, which increases the activities of key gluconeogenic enzymes due to stress [113]. WATCH, which is a multiplexed chemical sensor system, was used for the simultaneous detection of both cortisol and glucose, as well as for their continuous monitoring. To detect the biomarkers, cortisol aptamers and glucose oxidase enzymes were used. To evaluate long-term stress monitoring, they measured cortisol and glucose concentrations for a total of 8 h and 35 min in 10 individuals, analyzing how the concentration of each biomarker fluctuated in human sweat. The study also established a correlation between glucose and cortisol in sweat and demonstrated the potential use of glucose as a biomarker for stress.

Because of the lack of stress-related chemical biomarkers, we suggest glucose as an alternative stress-detecting chemical biomarker, considering the aforementioned studies. Martin et al. described a flexible epidermal microfluidic detection platform capable of the continuous and real-time monitoring of glucose and lactate levels in sweat, using oxidase enzymes [96]. This multiplexed platform for an efficient and fast sampling of sweat is fabricated through lithography and screen-printing. This platform is accessible as a stress-monitoring multiplexed chemical sensor, because lactate is another stress-related chemical biomarker. Chronic stress-induced epinephrine, which is a vital stress hormone, activates lactate dehydrogenase A to generate lactate and promotes the increase in lactate levels [114]. Moreover, lactate has almost 2–10 times higher concentrations in sweat than blood, having the potential to be utilized in a wearable monitoring sensor system [27,91,115]. Koh et al. reported epidermal microfluidic devices that can directly and reliably harvest sweat to measure pH, lactate, glucose, creatinine, and chloride, using colorimetric chemical assays based on enzymatic reactions [97]. As mentioned, lactate and glucose can be used as indicators to measure stress. The devices were able to detect each biomarker for up to 6 h in real-time monitoring, in daily life, and evaluated the capability of monitoring biomarkers. Moreover, creatinine is another chemical biomarker with the potential to be used for detecting stress, because of the exaggerated response of blood pressure to mental stress, which is associated with elevated plasmatic creatinine levels [116]. As such, when more biomarkers are measured by a multiplexed chemical sensor, a greater correlation to stress can be identified.

In addition to the lack of stress-related chemical biomarkers, continuous monitoring and sensing the low concentrations of chemical biomarkers in sweat are challenges in multiplexed chemical sensor systems. Mugo et al. introduced a flexible system targeting multiple biomarkers, by using electrochemical sensors, for the simultaneous detection of cortisol and pH in sweat, with a rapid detection within 1 min and reusability of up to 30 days [98]. To achieve a more stable system, the group utilized a molecularly imprinted polymer (MIP), a synthetic receptor, for detecting cortisol, instead of the traditional aptamer or antibody. This group demonstrated that both cortisol and pH are perceptive chemical biomarkers that can be used for indexing physiological stress. The introduced multiplexed chemical sensor was fabricated using a conductive microneedle as the substrate and two detection chambers, comprising a polyaniline layer for pH sensing and a cortisol molecularly imprinted polymer for cortisol sensing. Within 1 min, the pH-sensing chamber responded linearly to pH values in the range of 3.0–9.3, while the cortisol sensor exhibited linear changes within 0–100 ng/mL, with a limit of detection of 1.4 ± 0.3 ng/mL, which was evaluated at variable pH values in the 3.0–9.3 range. This multiplexed chemical sensor system can be used as a real-time stress monitoring device with superior linearity, based on its accurate detection of stress.

Using the multiplexed chemical sensor systems, stress can be measured by detecting cortisol and its correlation with other chemical biomarkers. Hence, the newly identified chemical biomarkers can correlate with stress levels even if they are at a low concentration in sweat.

## 4. Conclusions and Future Prospects

Mental stress can lead to severe illnesses, including chronic diseases; however, it is difficult to measure quantitively in daily life. Although, to monitor stress, conventional stress measurement systems mainly use physical sensors to detect a single biomarker, this approach is unsuitable for monitoring stress in real-time, and it is particularly difficult to quantify accurately because of crosstalk or artifacts, which physical biomarkers can be easily affected by. It is likely that chemical biomarkers are easily affected by the external environment; therefore, the biomarkers need to be validated and calibrated through compensating for each biomarker. Therefore, combinations of stress-related biomarkers should be measured to secure the more accurate monitoring of stress and to achieve the identification of the stress levels of individual through quantification. As a result, there are several possible combinations of physical and chemical sensors that can be used. Therefore, different combinations of physical and chemical sensors would be useful. Additionally, there are evident benefits and drawbacks to sensor combinations.

Chemical sensors, or combinations of chemical sensors, can quantitatively monitor changes in response to stress. However, in-the-state-of-art, the chemical biomarker-based sensors have difficulties of measuring stress in real-time. With the development of chemical sensors, stress-related hormones in body fluids such as cortisol can be quantitatively measured continuously, raising the possibility of using wearable devices for monitoring chemical biomarkers of stress [59]. However, the number of stress-related chemical biomarkers is insufficient, due to problems such as low concentrations, low accessibility, and influence from the surrounding environment. Therefore, to achieve high-performance stress monitoring, the system should be wearable, noninvasive, measurable in real-time, and integrated with various sensors capable of measuring multiple biomarkers. When integrating and configuring sensors, there are several restrictions and benefits in the systems that are currently being developed. For instance, chemical sensors have capabilities in terms of quantification, but are challenging to configure as wearable sensors, whereas physical sensors are simple to utilize as wearable sensors, but difficult to use to assess stress levels. When these sensors were integrated, challenges involving multiple sensor fabrication and crosstalk brought on by signal interference between the sensors needed to be taken into account. Physical sensors are well-developed into wearable platforms, but when measuring signals, the environment has a significant impact, and the outcome may vary depending on the signal-processing method [43]. This makes physical biomarkers challenging to apply in measuring stress levels. The currently commercialized watch-type wearable devices widely used today incorporate numerous physical sensors to ensure long-term use with high reliability. However, there is an increasing demand for the integration of chemical sensors to achieve the accurate and quantitative measurement of human stress. When using a chemical sensor, the measurement findings can be expressed quantitatively, but wearable platform development has not advanced very far, and non-invasive measurements are limited by the low concentration of biomarkers [26,27,28].

When building a multiplexed sensor, it is feasible to analyze the correlation of each biomarker to find novel biomarkers or algorithms, and cross-validate the responses of the physical and chemical sensors to ascertain whether the signal’s reaction is due to stress [105,106]. Additionally, the calibration errors brought on by environmental factors, such as ambient temperature or physical activity, allow for the continuous monitoring of stress levels [23,89,90,93]. However, there are several restrictions that need to be solved, such as challenges regarding material selection and the manufacturing process, as well as crosstalk between integrated sensors [30,91]. Proper stress assessment can only be done by overcoming this and optimizing the wearability of the device, reducing the stress that users may experience by reducing the size of the system and the discomfort of wearing.

Ultimately, it is anticipated that research on multiplexed stress monitoring sensors would increase in volume, to encompass not just quantitative stress monitoring, but also regarding a range of disorders associated with stress, if these limitations are first solved.

## Figures and Tables

**Table 1:** Representative biomarkers utilized to measure stress levels.

Variety of Signals (Abbr.)	Pathways (Signal Type)	Measured Locations	Analytical Methods	References
Electroencephalogram (EEG)	ANS (Physical)	Brain	Not standardized	[40,41,42,43,44]
Electrocardiogram (ECG), Respiration rate (RR)		Heart	Changes in R–R intervals in the QRS complex	[45,46,47]
Electromyogram (EMG)		Muscle	Not standardized	[48,49]
Electrodermal activity (EDA)		Skin	Changes in Amplitude and phase	[50,51,52]
Skin temperature (ST)		Skin	Changes in temperature	[53,54,55]
Cortisol	HPA axis (Chemical)	Body Fluid	Changes in cortisol concentration	[56,57,58,59]

**Table 2:** Multiplexed sensor systems and their characteristics.

Combinations of Multiplexed Sensor Systems	Combinations of Biomarkers	Characteristics					Ref.
		Location	Working Principles	Real-Time Monitoring *	Stress Monitoring *	Performance	
Physical sensors	GSR–ST	Wrist, shoulder	Calibration	O	O	GSR; SNR of 8.45 while walking ST; N/A	[83]
	ECG–GSR–ST	Chest	Validation	O	O	Overall, accuracy around 89% of human condition analysis	[84]
	ECG–RR–GSR	Chest, palm	Validation	O	O	Overall, accuracy up to 89% of stress level detection	[85]
	HRV–GSR–ST	Wrist	Validation	O	△	ST; sensitivity of 0.31 Ω/°C GSR; sensitivity of 0.28 μV/0.02 μS	[86]
	HR–RR	Chest	Validation	O	△	HR; accuracy of 97.6% (compared to single sensor) RR; accuracy of 93.8% (compared to single sensor)	[87]
	ECG–HR–ST	Chest	Validation	O	△	ECG; SNR of >20 dB HR; accuracy of 89% (compared to single sensor) ST; N/A	[88]
Physical-chemical sensors	ST–Glucose–Lactate	Forehead, wrist	Calibration	△ (Stabilized within 1 min)	△	ST; sensitivity of 0.18 %/°C Glucose; sensitivity of 2.35 nA/µM Lactate; sensitivity of 220 nA/mM	[89]
	ST–pH	Neck	Calibration	O	△	pH; sensitivity of 51.2 mV/pH ST; sensitivity of 0.85%/°C	[90]
	ECG–Lactate	Chest	Correlation	O	△	Lactate; sensitivity of 96 nA/mM ECG; N/A	[91]
	ECG–Lactate–pH	Ear	Correlation	O	△	pH; sensitivity of 50 mV/pH Lactate; sensitivity of 0.8 µA/mM ECG; SNR of 18 dB	[92]
	ST–PPG–Glucose	Forehead	Calibration Correlation	O	△	N/A	[23]
	BP–HR–Glucose–Lactate	Neck	Correlation	O	△	BP, HR; N/A Glucose; >100 mg/dL Lactate; N/A	[30]
	ST–Humidity–Glucose–pH	Wrist	Calibration	O	△	N/A	[93]
	ST–pH–Ammonium–Glucose–Lactate–Uric acid	N/A (only conducting animal-level studies)	Calibration Correlation	O	△	ST; sensitivity of 0.21%/°C pH; sensitivity of 59.7 mV/pH Ammonium; sensitivity of 59.7 mV/decade Glucose; sensitivity of 16.34 nA/mM Lactate; sensitivity of 41.44 nA/mM Uric acid; sensitivity of 189.60 nA/mM	[94]
Chemical sensors	Cortisol–Glucose	Arm	Correlation	O	△	Cortisol; sensitive in the range of 1–11 ng/mL Glucose; sensitive in the range of 1–10 mg/dl	[95]
	Glucose–Lactate	Arm and Lower back	Correlation	△ (reservoir was filled within 8 min from starting exercise)	△	Glucose; LOD of 50 μM Lactate; N/A	[96]
	pH–Lactate-Glucose–Creatinine	Lower back and volar forearm	Correlation	O (<1 min)	△	Glucose; LOD of 200 μM	[97]
	Cortisol–pH	Brow	Correlation	O (<1 min)	△	pH; LOD of 2 Cortisol; LOD of 1.4 ± 0.3 ng/mL	[98]
	Glucose–pH	Arm	Correlation	△ (sufficient sweat after 20 min)	△	Glucose; sensitivity of 10.89 μA/mM∙${\mathrm{cm}}^{2}$ pH; sensitivity of 71.44 mV/pH	[99]
	Cortisol–pH	Arm	Calibration	△ (reservoir was filled within 20 min from starting exercise)	△	pH; sensitivity of 69 mV/pH Cortisol; sensitive in the range of 1 pM to 1 uM, LOD of 0.2 pM	[100]

* Maturity and applicability of technology: O, Matured; △, Promising. Notes: GSR, galvanic skin response; ST, skin temperature; ECG, electrocardiogram; RR, respiration rate; HR, heart rate; HRV, heart rate variation; PPG, photoplethysmogram; BP, blood pressure.

## Data Availability

The data presented in this study are available from the corresponding authors upon request.
